# Gene-Polymorphism Effects on Growth Efficiency in the Kalmyk Breed of Central Asia

**DOI:** 10.3390/genes17010001

**Published:** 2025-12-19

**Authors:** Nurlybay Kazhgaliyev, Kaster Nurgulsim, Miras Gabbassov, Aizhan Makhanbetova, Assylbek Zhanabayev, Ascar Terlikbayev, Tolegen Assanbayev, Maxat Toishimanov, Tlekbol Sharapatov

**Affiliations:** 1 Faculty of Veterinary and Animal Husbandry Technology, S. Seifullin Kazakh Agrotechnical Research University, Zhenis Avenue, 62, Astana 010011, Kazakhstan; 2 Zootechnology and Veterinary Medicine, Faculty of Agriculture Science, Toraighyrov University, Lomov 64, Pavlodar 140008, Kazakhstan; 3 Food and Environment Safety Laboratory, Kazakh National Agrarian Research University, Almaty 050010, Kazakhstan; 4 Laboratory of Breeding and Biotechnology, Institute of Plant Biology and Biotechnology, Timiryazev 45, Almaty 050040, Kazakhstan

**Keywords:** *Kalmyk cattle*, CRTC2 gene, ELOVL6 gene, single nucleotide polymorphism, genotype, growth traits, meat quality, genetic markers, selective breeding

## Abstract

Background/Objectives: Understanding the genetic basis of growth and fat deposition is crucial for improving beef productivity in Kalmyk cattle, a breed well adapted to the extreme climatic conditions of Kazakhstan. The present study aimed to determine the effects of single-nucleotide polymorphisms (SNPs) in the CRTC2 and ELOVL6 genes on intramuscular fat content and to evaluate their associations with growth and meat quality traits in 18-month-old Kalmyk heifers raised under different environmental conditions. Methods: A total of 400 clinically healthy Kalmyk heifers (200 from LLP “Qazaq Asyldary” and 200 from LLP “Agrofirma Turikpen”) were examined. All animals originated from closed breeding herds, and only unrelated individuals without common ancestors to the third generation were included. Zootechnical measurements— live weight, withers height, chest depth, chest girth, and body length—were performed twice by a trained specialist. Backfat thickness and musculus longissimus dorsi depth were measured postmortem. Blood samples were collected for genomic DNA extraction using the GeneJET purification kit, and DNA quality was assessed by Nanodrop, Qubit, and agarose gel electrophoresis. Target fragments of CRTC2 and ELOVL6 were amplified (150–200 bp) and sequenced on an ABI 3500 system. SNP identification, allele frequencies, and genotyping were performed by alignment to the Bos taurus ARS-UCD1.2 reference genome. Statistical analyses were conducted in RStudio using linear and mixed models with “farm” as a random effect. Results: Only one informative polymorphism, g.133528A>G in ELOVL6, was detected. Three genotypes (AA, AG, GG) were observed, with the heterozygous AG genotype showing significantly higher live weight, greater body length, and improved linear measurements compared to AA and GG. No significant associations were detected with backfat thickness or muscle depth. The g.133528A>G polymorphism in ELOVL6 positively influences growth traits without increasing fatness, aligning with the naturally lean phenotype of Kalmyk cattle. Conclusions: The AG genotype may serve as a promising marker for selecting faster-growing animals in marker-assisted breeding programs.

## Simple Summary

Improving meat quality and increasing beef productivity are important tasks for modern animal breeding. In this study, we focused on Kalmyk cattle, a common beef breed in Central Asia. We investigated two specific genes that may influence fat accumulation and body growth. By analyzing genetic material from young heifers and comparing it with their growth indicators, we discovered that animals with one specific gene variant showed better weight gain and body development. These results help farmers identify and select the most productive animals at an early age. This can lead to more efficient breeding, better beef quality, and ultimately benefit both producers and consumers.

## 1. Introduction

Genetic polymorphisms that influence growth efficiency, energy metabolism, and lipid deposition represent key molecular factors that determine economically important traits in beef cattle, including live weight, average daily gain, carcass composition, and meat quality [1,2,3]. Candidate genes associated with metabolic regulation are increasingly used in marker-assisted and genomic selection, providing a foundation for developing more precise breeding strategies in beef production systems.

In the context of improving meat productivity, Kazhgaliev and Alibaev highlighted the importance of selection based on key phenotypic traits such as live weight, height at the withers, and coat length, supported by multi-trait index approaches. In Kazakh White-Headed cattle, selection is carried out in stages, beginning with identification of individuals exhibiting high phenotypic expression of these traits and followed by consistent improvement across all selection indicators [4]. These findings reinforce the relevance of combining traditional selection with molecular–genetic tools.

Among candidate genes with established roles in metabolism, *CRTC2* (CREB-regulated transcription coactivator 2) is recognized as a major regulator of energy homeostasis. *CRTC2* integrates signals from insulin, glucagon, AMPK, and PI3K/Akt pathways, influencing the transcription of genes involved in gluconeogenesis, lipogenesis, and myogenesis [5,6,7]. Its expression in liver, skeletal muscle, and adipose tissue highlights its importance for glucose turnover, mitochondrial activity, and adipocyte differentiation [6,7]. Variations within the *CRTC2* gene have been linked to differences in growth performance, intramuscular fat accumulation, and carcass characteristics in beef cattle [7,8]. In Qinchuan cattle, three SNPs (g.3001C>T, g.3034G>A, g.3467T>C) were associated with live weight, body size, subcutaneous fat thickness, and marbling, and favorable haplotypes were shown to enhance fattening efficiency and meat quality [7,8,9].

Another candidate gene of interest, *ELOVL6* (elongation of very long-chain fatty acids 6), encodes a key enzyme involved in the elongation of long-chain fatty acids, thereby influencing lipid composition and fat deposition in bovine tissues [10,11]. Functional studies demonstrated that overexpression of *ELOVL6* alters the fatty acid profile by increasing the levels of stearic and arachidonic acids and reducing palmitic and myristic acids, which contributes to desirable sensory attributes of beef, including improved texture and marbling [11,12,13].

The *DGAT1* gene, widely studied in beef and dairy breeds, is known to influence intramuscular fat content and subcutaneous fat thickness. The K allele of the K232A polymorphism is associated with enhanced marbling and increased fat deposition in breeds such as Hereford, Angus, and zebu populations, although its effect varies among genetic backgrounds [14,15].

In Kazakhstan, the genetic resources of beef cattle have been expanded through the use of Kazakh Akbas, Auliekol, and Kalmyk breeds, the latter being the most widespread due to its adaptability, robust constitution, and stable productivity. Currently, the population of *Kalmyk cattle* in the country exceeds 63,720 head, representing 8.8% of the national beef cattle herd. Despite their adaptability, *Kalmyk cattle* exhibit lower intramuscular fat content and limited marbling compared to specialized commercial breeds, which constrains their economic value under modern beef production systems [16].

Existing studies indicate a lack of comprehensive data on the functional significance of polymorphic genes controlling fat deposition, growth performance, and carcass traits in *Kalmyk cattle*. In particular, associations between specific genotypes and body size, carcass characteristics, subcutaneous fat thickness, intramuscular fat levels, and eye muscle area remain poorly defined [17,18,19]. This gap limits opportunities for implementing marker-assisted selection aimed at improving meat quality traits in this indigenous breed.

Given these limitations, investigating polymorphisms in key metabolic genes represents an important step toward enhancing the genetic potential of *Kalmyk cattle* under the production conditions of Kazakhstan. Such research may provide valuable insights for improving growth efficiency, fat deposition patterns, and overall meat quality.

The aim of the study was to determine the effects of single-nucleotide polymorphisms (SNPs) in the *CRTC2* and *ELOVL6* genes on intramuscular fat content and to evaluate their associations with growth and meat quality traits in *Kalmyk cattle* raised under different environmental conditions in Kazakhstan.

## 2. Materials and Methods

### 2.1. Animals and Research Site

**Figure 1:**
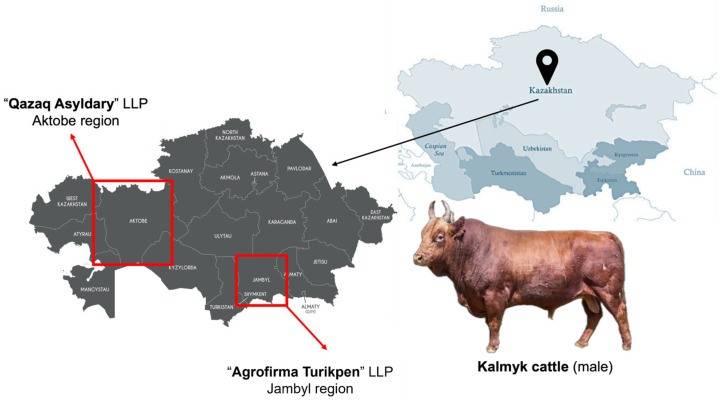
Location of research site: (1) Aktobe region “Qazaq Asyldary” LLP and (2) Jambyl region “Agrofirma Turikpen” LLP, Kazakhstan with external phenotype of *Kalmyk cattle*.

A total of 400 clinically healthy Kalmyk heifers aged 18 months were included in the study, with 200 animals sampled from LLP “Qazaq Asyldary” (Aktobe region) and 200 from LLP “Agrofirma Turikpen” (Zhambyl region). Both farms maintain closed nucleus breeding herds, and only unrelated animals without common ancestors up to the third generation were selected for analysis. The sampled animals represented the major intra-breed lines maintained at these farms. Despite regional differences, feeding regimes, housing conditions, veterinary services, and overall management practices were standardized, which minimized the environmental influence on phenotypic traits (Figure 1).

All laboratory procedures were carried out at the Molecular Genetics Laboratory of S. Seifullin Kazakh Agrotechnical Research University.

### 2.2. Zootechnical and Genetic Analysis

Zootechnical measurements were performed according to the standard breed-specific protocols in the morning prior to feeding. Live weight was recorded after an overnight fast using calibrated electronic scales. Linear body measurements—including withers height, chest depth, chest girth, and oblique body length—were taken twice by a trained specialist who was blinded to the genotypes. The mean values obtained from the two measurements were used for analysis. Subcutaneous fat thickness and the depth of the musculus longissimus dorsi were assessed postmortem using a metal measuring ruler at the level of the 12th–13th rib. Inter-farm differences were accounted for in the subsequent statistical analysis.

### 2.3. DNA Extraction and Quality Control

For each animal, 8–10 mL of whole blood were collected from the jugular vein into vacuum tubes containing EDTA as an anticoagulant. The samples were transported on ice and stored at −20 °C until further processing. Genomic DNA was extracted using the GeneJET Genomic DNA Purification Kit (Thermo Fisher Scientific Inc., Waltham, MA, USA). DNA quality was assessed using Nanodrop spectrophotometry, Qubit fluorometric quantification, and electrophoresis on a 1% agarose gel, with only samples exhibiting an A260/280 ratio of 1.8–2.0 accepted for downstream analyses.

### 2.4. Phenotypic Measurements

Phenotypic data included live weight and linear body measurements. All measurements were taken twice by a specialist who was blinded to the animals’ genotypes. These traits were used to assess growth, development, and meat productivity, and were subsequently compared with the animals’ genotypes [20].

### 2.5. Genotyping and SNP Analysis

Purified amplicons were sequenced on an ABI 3500 Genetic Analyzer using the BigDye Terminator v3.1 Cycle Sequencing Kit (Thermo Fisher Scientific Inc., Waltham, MA, USA). Sequencing quality was evaluated based on Q30 metrics and visual inspection of chromatograms. Sequence analysis was performed by aligning reads to the Bos taurus ARS-UCD1.2 reference genome. Single-nucleotide polymorphisms (SNPs) were identified, allele and genotype frequencies were calculated.

### 2.6. PCR Amplification

Amplification of the target fragments of the CRTC2 and ELOVL6 genes was performed using gene-specific primers that produced amplicons of 150–200 bp in a Bio-Rad T100 thermal cycler (Bio-Rad Laboratories, Inc., Hercules, CA, USA). Annealing temperatures ranged from 58 to 60 °C. Amplicon specificity was confirmed electrophoretically.

### 2.7. Data Processing

Primary data organization was performed in Excel; however, all statistical analyses were conducted in RStudio (version 2023.06). To assess the effects of genotypes, linear and mixed linear models were applied, with “farm” included as a random effect. Association results were interpreted considering confidence intervals, inter-farm variability, and potential environmental influences.

## 3. Results

To evaluate the effects of *CRTC2* and *ELOVL6* genotypes on body size and growth, a cohort of 18-month-old Kalmyk heifers was examined. Comparative analysis of body conformation included the principal linear measurements (oblique body length, withers height, chest girth, chest depth) as well as live weight. These indicators were used to assess the overall development, uniformity, and compliance of the animals with breed standards.

The descriptive statistics for each trait are presented in Table 1, including the mean (X ± Sx), standard deviation (σ), and coefficient of variation (CV%). Across both farms, the heifers demonstrated favorable exterior characteristics, with mean values exceeding the first-class standard for live weight.

Analysis of body measurements demonstrated that animals from both farms exhibited robust development and were consistent with the Kalmyk breed type standard. Slightly higher absolute values for oblique body length, withers height, and chest depth were observed, suggesting a strong skeletal structure and good adaptability to diverse management conditions. The consistently low coefficients of variation (CV < 5% for most traits) indicate a high degree of herd uniformity, which is advantageous for selection. Notably, average live weight was 334.3 kg and 343.2 kg in the two herds, representing increases of 4.4% and 7.2% above the first-class standard (320 kg).

### 3.1. Molecular Genetic Analysis

The molecular genetic stage of the study focused on identifying polymorphic loci in the CRTC2 and ELOVL6 genes that may be associated with performance traits in *Kalmyk cattle*. In the first phase, four primer pairs were designed from the available nucleotide sequences of *CRTC2* (NM_001076250.1) and *ELOVL6* (NM_001102155.1) using the Primer-BLAST tool (NCBI) (version 2.5.0). Amplicon lengths were 205 bp, 529 bp, 240 bp, and 759 bp, selected to cover coding regions with potential functional significance (Table 1).

**Figure 2:**
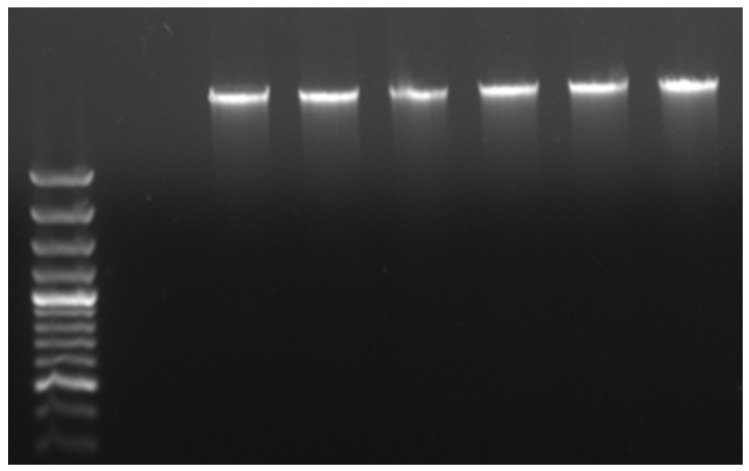
Electrophoresis analysis confirming the quality of DNA extracted from blood samples of *Kalmyk cattle*.

Genomic DNA was extracted from blood samples using the GeneJET Genomic DNA Purification Kit (Thermo Fisher Scientific Inc., Waltham, MA, USA). DNA yield and purity were assessed spectrophotometrically (A260/A280 ratio of 1.8–2.0), and integrity was confirmed by 1% agarose gel electrophoresis, which showed distinct, high-molecular-weight bands without degradation (Figure 2) [20,21]. These results demonstrated that the isolated DNA was of sufficient quality and concentration for subsequent PCR amplification and sequencing.

**Figure 3:**
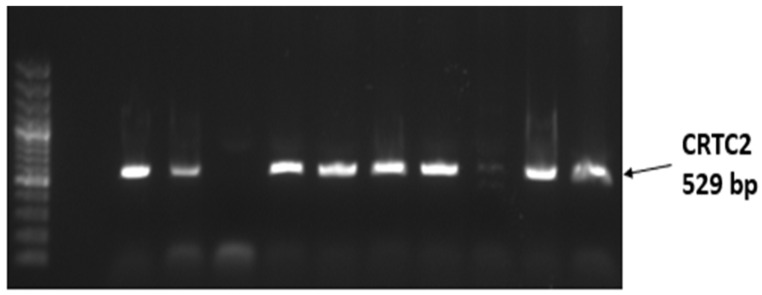
Electrophoretic detection of the 529 bp PCR product amplified from the *CRTC2* gene.

Polymerase chain reactions were performed with DreamTaq Hot Start PCR Master Mix (2X) (Thermo Scientific, USA) under a standard thermal cycling profile (denaturation, annealing, extension). Among the tested primer sets, only one pair for *CRTC2* successfully amplified the target fragment, producing a 529 bp product (Figure 3). Two primer sets (one for *CRTC2*, one for *ELOVL6*) failed to amplify, likely due to suboptimal primer design or sequence mismatches in the Kalmyk genome. These results indicate that reliable amplification was achieved for specific loci, providing a basis for subsequent sequencing and polymorphism detection.

### 3.2. SNP Analysis with Primer Sets

In the initial stage of SNP analysis, four primer pairs were designed for the *CRTC2* and *ELOVL6* genes (Table 2). Among these, only one primer pair for *CRTC2* successfully amplified the target fragment, producing a 529 bp product. Sequencing of this region showed no detectable polymorphisms in the *Kalmyk cattle* population, indicating that the locus was monomorphic. Consequently, additional primer sets were developed to expand the coverage of both candidate genes and to explore other potentially polymorphic regions.

New primer sequences were generated for *CRTC2* (729 bp) and *ELOVL6* (292 bp, 308 bp, 176 bp, 497 bp, 325 bp, 339 bp) using the Primer-BLAST tool. The primer designs targeted coding and regulatory regions of functional importance. The specific sequences were as follows:

- *CRTC2*:

F: GGCGGTCTTGGAAGAGTTCA (729 bp)/R: CTCTGGCTCTCTCCTCCACT (729 bp)

- *ELOVL6*:

F1: TCAGGGCGTGTTTCTCATTGC/R1: ACTGGGAGAAACGCATAC (292 bp)

F2: CCATCATCTCTTCAGGGCGTG/R2: AGGATACTGGGAGAAACGCA (308 bp)

F3: ATGTTGCCAAATGGACTC/R3: CAGAAAAACAAAATCTCA (176 bp)

F4: TCTCTTTAGGGAAGGGGGCA/R4: AGAGGAGATGGCCTGAGTGT (497 bp)

F5: TGAAGCCTATCGGAGAAT/R5: CAGATCAAACCTCCAGAT (325 bp)

F6: CTGCCCCACTATGCTGCAAT/R6: AGACAGTTAGAAGGATGGGAGTAAA (339 bp)

**Figure 4:**
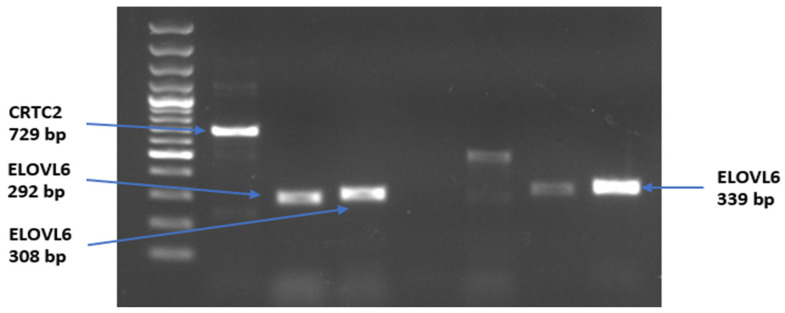
PCR amplification results of selected primer sets for the *CRTC2* and *ELOVL6* genes in *Kalmyk cattle*.

During the second phase of the study, PCR testing with these newly designed primers showed that four primer pairs successfully amplified their respective loci: *CRTC2* (729 bp) and *ELOVL6* (292 bp, 308 bp, 339 bp). Other primer sets failed to yield specific products, most likely due to sequence mismatches in the Kalmyk genome or limitations in primer design. The successful amplicons (Figure 4) provided suitable fragments for sequencing and subsequent polymorphism analysis.

**Figure 5:**
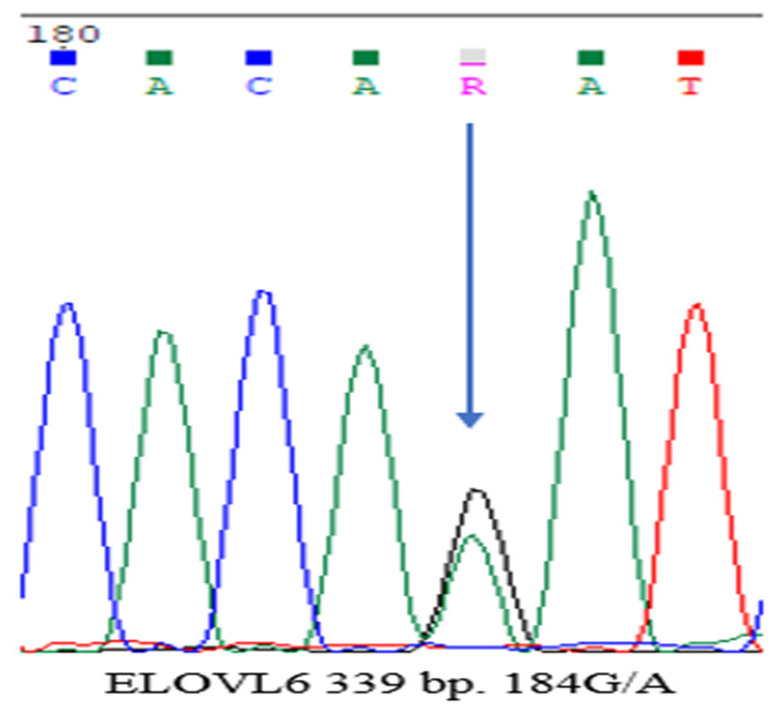
Representative genotyping results obtained using the 339 bp primer targeting the *ELOVL6* gene in *Kalmyk cattle*.

Genotyping was then performed on these four amplified regions. Among them, a single polymorphism was identified in the 339 bp fragment of the *ELOVL6* gene: g.133528A>G (184G/A) (Figure 5). No mutations were observed in the other amplified regions.

To assess the functional relevance of this polymorphism, association analysis was conducted using data from 400 Kalmyk heifers (18 months old). Phenotypic traits considered included live weight, oblique body length, withers height, chest depth, chest girth, subcutaneous fat thickness, and longissimus dorsi muscle depth. Statistical analyses were carried out in RStudio, applying standard models for genotype–phenotype association.

### 3.3. Polymorphism Analysis of the ELOVL6 Gene and Association with Growth Traits

In this study, particular emphasis was placed on detecting polymorphic loci within the *ELOVL6* gene, which encodes an enzyme responsible for the elongation of long-chain fatty acids. This gene plays a central role in lipid metabolism and is therefore directly linked to the development of meat quality traits in *Kalmyk cattle*. Four primer pairs covering different regions of the gene were designed and tested. Among them, only the 339 bp amplicon (primer F6/R6) was found to be informative, while other fragments (292 and 308 bp) were monomorphic and excluded from further analysis.

Genotyping of the 339 bp amplicon was carried out in two stages: PCR product purification using ExoSAP-IT^TM^ (Thermo Scientific, USA) followed by sequencing with the BigDye Terminator v3.1 kit (Applied Biosystems, USA). Sequence analysis revealed a single-nucleotide polymorphism (SNP), g.133528A>G (184G/A), previously described in international studies as a variant with potential biological significance (Table 3). Based on this SNP, three genotypic classes were identified: AA (homozygote for allele A), AG (heterozygote), and GG (homozygote for allele G).

The linear regression analysis demonstrated that SNP genotypes exerted varying effects on the studied morphometric traits, with the most pronounced associations observed for live weight and body diagonal length (Table 4).

The estimated mean live weight for individuals carrying the AA genotype was 338.1 ± 1.84 kg, representing the baseline phenotype. Animals with the AG genotype exhibited a significant increase of 13.0 ± 2.64 kg relative to the AA group (*p* = 1.79 × 10^−6^), indicating a strong positive association between the heterozygous genotype and body mass. Conversely, the GG genotype displayed a smaller, statistically non-significant increase of 2.6 ± 2.52 kg (*p* = 0.302 *), suggesting a possible dominance effect of the A allele.

The AA genotype showed a mean body diagonal length of 137.5 ± 0.86 cm. The AG genotype was associated with a significant elongation of 4.15 ± 1.23 cm (*p* = 0.0009 *), whereas the GG genotype demonstrated a modest, non-significant increase of 1.55 ± 1.18 cm (*p* = 0.189 *). This pattern reinforces the beneficial influence of the heterozygous genotype on overall body frame development.

Cattle with the AA genotype had an average withers height of 120.3 ± 0.33 cm. Both AG and GG genotypes showed small increases of 0.29 ± 0.47 cm and 0.74 ± 0.45 cm, respectively; however, neither effect was statistically significant (*p* > 0.05 *), suggesting that this trait is relatively insensitive to the allelic substitution at the studied locus.

The baseline chest depth for the AA genotype was 56.0 ± 0.54 cm. The AG genotype exhibited an increase of 1.39 ± 0.77 cm, approaching significance (*p* = 0.071 *), whereas the GG genotype showed a non-significant increment of 0.93 ± 0.73 cm (*p* = 0.206 *). The near-significant effect in heterozygotes may reflect a partial additive influence on thoracic development.

The AA genotype group presented a mean chest girth of 124.4 ± 0.73 cm. Slight reductions were observed in AG (−0.14 ± 1.05 cm) and GG (−1.32 ± 1.00 cm) genotypes; however, these effects were not statistically significant (*p* > 0.05 *), indicating minimal genotype influence on this dimension of body conformation.

Baseline backfat thickness among AA homozygotes was 0.74 ± 0.01 cm. Both AG and GG genotypes showed marginal reductions (−0.025 ± 0.019 cm and −0.021 ± 0.018 cm, respectively), neither of which reached statistical significance (*p* > 0.05 *). Thus, this SNP appears unrelated to subcutaneous fat deposition.

The AA genotype exhibited a mean muscle thickness of 3.44 ± 0.02 mm. Negligible decreases were recorded for AG (−0.024 ± 0.023 mm) and GG (−0.024 ± 0.022 mm) genotypes, with no statistical support for genotypic differentiation (*p* > 0.05 *).

Overall, the heterozygous AG genotype consistently exhibited favorable phenotypic values for growth-related characteristics, particularly live weight and body diagonal length, suggesting a potential heterosis effect or partial dominance at this locus. Other morphometric parameters, including withers height, chest girth, backfat, and muscle thickness, were largely unaffected, indicating that this SNP primarily modulates overall body size rather than shape or tissue composition.

**Figure 6:**
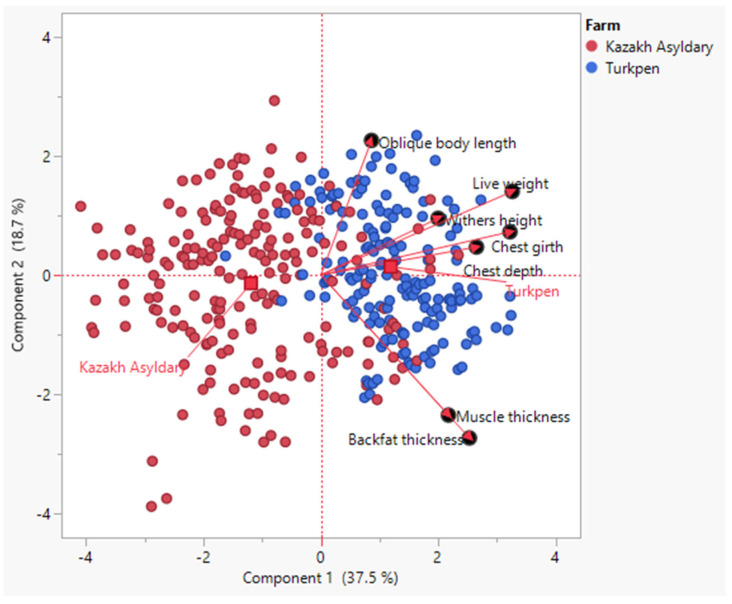
Principal Component Analysis (PCA) of growth and body measurement traits in Kalmyk heifers from two farms (“Qazaq Asyldary” and “Agrofirma Turkpen”).

Principal component analysis (PCA) was performed to summarize variation in morphometric and growth traits of Kalmyk heifers with the first two principal components explaining 56.2% of the total variance (PC1: 37.5%; PC2: 18.7%) as shown in Figure 6. Heifers from the Kazakh Asyldary and Turkpen farms formed two partially overlapping but distinct clusters, reflecting differences in herd structure and management.

Live weight, oblique body length, withers height, chest girth, and chest depth loaded strongly and positively on PC1, indicating that this axis represents overall body size and growth performance. In contrast, muscle thickness and subcutaneous fat thickness were more strongly associated with PC2, distinguishing individuals with greater carcass tissue development.

Heifers from Turkpen tended to cluster toward higher values on PC1, consistent with superior growth-related traits, while those from Kazakh Asyldary showed greater dispersion across both axes, reflecting more within-herd variability. These results suggest that farm management and breeding practices influenced body conformation and growth performance, with Turkpen animals exhibiting more uniform development across key growth traits.

## 4. Discussion

The present study provides evidence that the g.133528A>G polymorphism of the ELOVL6 gene is associated with growth-related traits in Kalmyk heifers, while no significant associations were detected for fatness traits. These findings are relevant given the limited molecular–genetic characterization of the Kalmyk breed and the need to clarify the biological background of its performance traits [20].

Heifers carrying the heterozygous AG genotype displayed significantly greater live weight and oblique body length than AA animals (*p* < 0.001). In contrast, the GG genotype showed only small and statistically non-significant increases in these traits. Although this pattern appears to be non-additive, such interpretations should be made cautiously because the study did not report genotype and allele frequencies, Hardy–Weinberg equilibrium testing, or statistical models adjusting for farm effects. Without these elements, it is not possible to distinguish genetic effects from potential environmental or management influences.

The absence of statistically significant associations between the polymorphism and fatness indicators—including backfat thickness and longissimus dorsi muscle depth—aligns with the naturally lean phenotype of *Kalmyk cattle*. However, this finding must also be interpreted with caution, as the statistical model did not account for farm differences, feeding regime, or slaughter age. These factors can mask small genotype-driven differences in fat deposition. Thus, the lack of association should not be seen as definitive evidence that ELOVL6 variation has no functional relevance to fatness traits in this population. The biology of *ELOVL6* helps explain why this might be the case. The gene encodes an enzyme that extends fatty acids from 16 to 18 carbons, a key step in fat metabolism. Shifts in *ELOVL6* activity can change the balance of fatty acids in fat tissue more stearic acid and long-chain unsaturated fats, less palmitic acid. These shifts can soften fat and alter meat quality, but they may also influence how animals use energy. If the G allele enhances *ELOVL6* activity, it could tilt the metabolism toward using nutrients more efficiently for muscle growth instead of storing them as fat. The G allele appears to confer an advantage when present in one copy, but its benefit plateaus in homozygous form, or may be moderated by other physiological constraints. Such overdominance, while relatively uncommon for single-gene traits, has been reported in livestock where heterozygotes display superior fitness or growth performance compared with either homozygote. Further molecular work (e.g., comparing ELOVL6 mRNA/protein levels or fatty acid profiles in AA vs. AG vs. GG animals) would be needed to confirm these hypotheses about mechanism [22].

Comparison with other breeds highlights the role of breed-specific genetic architecture. In Qinchuan cattle, which have undergone selection for marbling, polymorphisms in the CRTC2 gene (g.3001C>T, g.3034G>A, g.3467T>C) have been reported to significantly influence not only growth traits but also subcutaneous fat thickness and intramuscular fat content [7,23,24]. This is consistent with the broader metabolic regulatory functions of CRTC2. By contrast, ELOVL6 performs a more specialized biochemical function— fatty acid chain elongation— which may influence fat composition more than fat quantity. As a result, the phenotypic effects of ELOVL6 polymorphisms in breeds with limited fat variability, such as *Kalmyk cattle*, may remain subtle.

Findings from major-effect genes such as DGAT1 further illustrate this point. The K232A variant of DGAT1 strongly affects intramuscular and subcutaneous fat deposition in specialized beef and dairy breeds, including Angus and Hereford [25]. In our study, the ELOVL6 g.133528A>G variant did not exhibit comparable effects, indicating that it is not a major lipogenic driver in *Kalmyk cattle*. These observations agree with earlier reports highlighting the limited evidence linking polymorphic variants to fatness traits in this breed [26].

Age and production conditions also help explain the absence of fatness associations. Studies in breeds selected for marbling typically evaluate cattle at slaughter under high-energy diets, which maximizes the expression of genetic differences in lipid deposition. In contrast, the animals in the present study were evaluated at 18 months of age, prior to intensive finishing. Consequently, genotype-specific effects on fat traits may emerge only later under more energy-dense feeding conditions [27].

It is also useful to compare these findings with other local breeds. The Kazakh Akbas (Kazakh White-Headed) and Auliekol breeds are Kazakhstani beef cattle that have been increasingly utilized in cross-breeding and herd improvement programs [28]. These breeds are generally robust and well adapted to extensive production systems, but they generally exhibit higher fat deposition than Kalmyk breed. However, studies explicitly examining *ELOVL6* polymorphisms in those breeds are limited. Our results in Kalmyk cattle therefore provide a valuable reference point and emphasize that the impact of candidate genes must be validated across diverse genetic backgrounds before general conclusions can be drawn [29,30].

The observation that heterozygous AG animals showed the strongest growth performance has important physiological implications. One possibility is that AG animals achieve an intermediate level of *ELOVL6* activity, creating a more balanced fatty acid profile. This balance could enhance membrane composition or cellular signaling in muscle and adipose tissue, thereby supporting more efficient nutrient utilization. For example, if the G allele promotes greater synthesis of stearic and oleic acids then fatty acids considered metabolically favorable and heterozygotes might benefit from improved insulin sensitivity and muscle growth. By contrast, AA homozygotes, with potentially lower *ELOVL6* activity, could accumulate more palmitic acid, which has been linked to reduced metabolic efficiency. GG homozygotes, although expected to have the highest *ELOVL6* activity, may experience diminishing returns, with excess elongation or compensatory metabolic adjustments limiting further gains.

Despite these limitations, the consistent association between the AG genotype and enhanced growth performance is noteworthy. Greater body weight and larger frame size at 18 months—combined with the absence of increased fat deposition—may offer practical advantages for production efficiency. However, before the AG genotype can be considered a useful marker for selection, further research is required to evaluate its effects at slaughter age and its relationship with carcass and meat quality traits, including fatty acid profile and marbling [31].

It is also possible that the g.133528A>G polymorphism is in linkage disequilibrium with another functional variant within ELOVL6 or a neighboring gene, which could partly explain the observed phenotype [32,33]. This possibility underscores the need for future molecular analyses.

In summary, the ELOVL6 g.133528A>G polymorphism appears to influence growth-related traits in Kalmyk heifers but shows no detectable effect on fatness traits under the conditions of this study. Further research incorporating larger sample sizes, mixed-effect statistical models, and carcass-quality evaluations is needed to determine the potential suitability of this locus for marker-assisted selection.

## 5. Conclusions

From a breeding perspective, the AG genotype emerges as a promising marker for selecting faster-growing, larger-framed animals that retain carcass leanness. Such outcomes are economically advantageous, as they improve feed efficiency and market weights without incurring the inefficiency of early fat deposition. At the same time, the results highlight the breed-specific nature of genetic effects: unlike other fat-related genes such as *CRTC2* or *DGAT1*, *ELOVL6* variation in *Kalmyk cattle* appears to influence growth rather than fatness. This underscores the importance of validating candidate markers across different breeds and production systems before applying them broadly.

Ultimately, the g.133528A>G variant in *ELOVL6* provides novel insight into genetic determinants of growth in *Kalmyk cattle* and supports its potential utility in marker-assisted selection. These findings suggest that animals carrying the AG genotype can be selected within the herd and used in breeding programs. Such research outcomes can facilitate the early selection of promising beef cattle and inform decisions on the use of animals with favorable genotypes in selective breeding processes.

## Figures and Tables

**Table 1:** Body dimensions (cm) and live weight (kg) of Kalmyk breed heifers (18 months old).

Indicators	«Kazakh Asyldary» LLP (*n* = 200)			“Agrofirma Turikpen” LLP (*n* = 200)		
	Mean	σ	Cv, %	Mean	σ	Cv, %
A live weight, kg	334.29 ± 2.53	11.40	3.97	343.18 ± 2.66	15.07	4.11
Oblique body length, cm	136.94 ± 0.67	3.82	2.89	138.75 ± 0.58	3.30	2.48
Height at withers, cm	120.81 ± 0.54	3.04	2.52	121.32 ± 0.26	1.54	1.22
Chest girth, cm	123.8 ± 0.65	3.67	2.98	125.22 ± 0.83	4.70	3.35
Chest depth, cm	56.78 ± 0.37	2.09	2.20	57.16 ± 0.52	2.95	4.83
Subcutaneous fat thickness, cm	0.76 ± 0.01	0.07	9.64	0.81 ± 0.01	0.06	7.06
Muscle eye thickness, cm	3.43 ± 0.01	0.11	3.34	3.53 ± 0.01	0.06	7.04
Live weight standard, kg	320	—	—	320	—	—

Standard deviation (σ) and coefficient of variation (CV%).

**Table 2:** Primer sequences of CRTC2 and ELOVL6 genes.

Primer	Sequence (5′-3′)	Fragment Size	Tm/°C
CRTC2 F1 CRTC2 R1	TTCATCTCGGAGCACTCGAC TCGAAACAACTGTCCTTCTCT	205 bp	58.93
CRTC2 F2 CRTC2 R2	GTGGCTTGTATTGGGAGGGT AGTCGAAACAACTGTCCCTTCT	529 bp	59.90
ELOVL6 F1 ELOVL6 R1	CGTAGCGACTCCGAAGATCA CTGCAAGGGTCAGAGACCAG	240 bp	57.65
ELOVL6 F2 ELOVL6 R2	AAGATCAGCCCCAGTGAACA AAGTGGCAGAAGAGCACGAA	759 bp	58.05

**Table 3:** SNP locus in the gene sequence of the ELOVL6 gene NC_037333.1, F6/339 bp primer (marked in red).

Primer	Gene Sequences
ELOVL6 F6/339 bp NC_ 037333.1	accactaccc catggcaaat tgaagcctat cggagaattg actgccacat agataatcctgctgtcattt aacattccag aaaaacttgg gcaggcaagt ttttatagct gccccactatgctgcaatag atttcctttc tcttgaagat tttccactgc acttcaattc cagtatcgtgaaaacattcc tggattgaaa aagtttttta aaccaaatct tcatttcaga cataaatcatgcaatttgat acacaaggtc tgagcatcaa cccaagtcta tgagtttgca gtgaaaaacattctaatgag aaatgctctg ttcacaaatc aggaggtttg atctggacct tctagtttgctcatgagaac agagccacta ctatattcaa aaatcatgta ttgaggtgat tattccagttaatttactcc catccttcta actgtctgtt cccttctcca ggggatcttc ccgacccagggatcaaaccc gcatctccca catttcaggc agattgttta ccatctgagc caccagggaa

**Table 4:** Association of Genotypes with Phenotypic Variations in *Kalmyk cattle*.

Phenotype	Genotype	Estimate	Std. Error	t Value	*p*-Value	Explanation
Live weight (kg)	(Intercept)	338.138	1.838	183.956	<2 × 10^−16^	Baseline weight when all SNP variables are zero (AA genotype)
	SNPAG	13.009	2.642	4.924	1.79 × 10^−6^	Weight increases by 13.009 kg in AG genotype compared to AA (statistically significant)
	SNPGG	2.605	2.519	1.034	0.302	Weight increases by 2.605 kg in GG genotype compared to AA (not statistically significant)
Body diagonal length (cm)	(Intercept)	137.477	0.859	160.111	<2 × 10^−16^	Baseline diagonal length (AA genotype)
	SNPAG	4.146	1.234	3.360	0.000937	Length increases by 4.146 cm in AG genotype compared to AA (statistically significant)
	SNPGG	1.550	1.177	1.317	0.189	Length increases by 1.550 cm in GG genotype compared to AA (not statistically significant)
Withers height (cm)	(Intercept)	120.339	0.326	369.200	<2 × 10^−16^	Baseline height (AA genotype)
	SNPAG	0.285	0.468	0.607	0.544	Height increases by 0.285 cm in AG genotype compared to AA (not statistically significant)
	SNPGG	0.743	0.447	1.662	0.098	Height increases by 0.743 cm in GG genotype compared to AA (not statistically significant)
Chest depth (cm)	(Intercept)	56.015	0.535	104.782	<2 × 10^−16^	Baseline chest depth (AA genotype)
	SNPAG	1.395	0.768	1.815	0.0711	Chest depth increases by 1.3945 cm in AG genotype compared to AA (marginal significance)
	SNPGG	0.931	0.733	1.270	0.206	Chest depth increases by 0.9306 cm in GG genotype compared to AA (not statistically significant)
Chest girth (cm)	(Intercept)	124.400	0.729	170.547	<2 × 10^−16^	Baseline chest girth (AA genotype)
	SNPAG	−0.138	1.048	−0.131	0.896	Chest girth decreases by 0.1377 cm in AG genotype compared to AA (not statistically significant)
	SNPGG	−1.319	1.000	−1.319	0.189	Chest girth decreases by 1.3189 cm in GG genotype compared to AA (not statistically significant)
Backfat thickness (cm)	(Intercept)	0.738	0.013	54.932	<2 × 10^−16^	Baseline backfat thickness (AA genotype)
	SNPAG	−0.0255	0.019	−1.320	0.188	Backfat thickness decreases by 0.0255 cm in AG genotype compared to AA (not statistically significant)
	SNPGG	−0.0209	0.018	−1.134	0.258	Backfat thickness decreases by 0.0209 cm in GG genotype compared to AA (not statistically significant)
Muscle thickness (mm)	(Intercept)	3.443	0.016	217.210	<2 × 10^−16^	Baseline muscle thickness (AA genotype)
	SNPAG	−0.0239	0.023	−1.050	0.295	Muscle thickness decreases by 0.02393 mm in AG genotype compared to AA (not statistically significant)
	SNPGG	−0.0241	0.022	−1.110	0.269	Muscle thickness decreases by 0.02410 mm in GG genotype compared to AA (not statistically significant)

## Data Availability

The original contributions presented in this study are included in the article. Further inquiries can be directed to the corresponding authors.

## Abbreviations

The following abbreviations are used in this manuscript:

CRTC2	CREB-Regulated Transcription Coactivator 2
CREB	cAMP Response Element-Binding Protein
ELOVL6	Elongation of Very Long-Chain Fatty Acids 6
SNP	Single-Nucleotide Polymorphism
PCR	Polymerase Chain Reaction
qRT-PCR	Quantitative Real-Time Polymerase Chain Reaction
PEPCK	Phosphoenolpyruvate Carboxykinase
G6Pase	Glucose-6-Phosphatase
AMPK	AMP-Activated Protein Kinase
DNA	Deoxyribonucleic Acid
